# High rate of attention deficit hyperactivity disorder among children 6 to 17 years old in Southwest Ethiopia findings from a community-based study

**DOI:** 10.1186/s12888-023-04636-9

**Published:** 2023-03-08

**Authors:** Kemal Aliye, Elias Tesfaye, Matiwos Soboka

**Affiliations:** 1 College of Health and Medical Sciences, Department of psychiatry, Haramaya University, Haramaya, Ethiopia; 2 Institute of health, Facult of medicine, Department of psychiatry, Jimma University, Jimma, Ethiopia

**Keywords:** Attention deficit hyperactivity disorder, Children, Jimma, Southwest Ethiopia

## Abstract

**Background:**

Attention-deficit/hyperactivity disorder is among the common neuropsychiatric disorders affecting children and adolescents. The disorder affects the life of children, their parents, and the community when left untreated. Although evidence indicated a high prevalence of attention-deficit/hyperactivity disorder in the developed world, there is limited evidence in developing countries, particularly, Ethiopia. Therefore, this study aimed to determine the prevalence and associated factors of attention deficit hyperactivity disorder among Ethiopian children aged 6 to 17 years.

**Methods:**

A community-based cross-sectional study was conducted from August to September 2021 among children aged 6 to 17 years in Jimma town. A multistage sampling technique was applied to select 520 study participants. Data were collected by using the Vanderbilt Attention Deficit Hyperactivity Disorder- Parent Rating scale as a modified, semi-structured, and face-to-face interview. The association between independent variables and the outcome variable was investigated using bi-variable and multivariable logistic regression. The final model level of significance was set at a p-value of < 0.05.

**Result:**

A total of 504 participants were involved in the study with a response rate of 96.9%. The overall prevalence of attention deficit hyperactivity disorder in this study was (9.9%, n = 50). Maternal complication during pregnancy (Adjusted odds ratio (AOR) = 3.56, 95% CI = 1.44–8.79, mothers illiteracy (AOR = 3.10, 95% CI = 1.24–7.79), attending primary school (AOR = 2.97, 95% CI = 1.32–6.73), history of head trauma (AOR = 3.20, 95% CI = 1.25–8.16), maternal alcohol use during pregnancy (AOR = 3.54, 95% CI = 1.26-10), bottle feeding during first six months (AOR = 2.87, 95% CI = 1.20–6.93) and child’s age 6–11 years (AOR = 3.86, 95% CI = 1.77–8.43) were significantly associated with attention deficit hyperactivity disorder.

**Conclusion:**

In this study, one in ten children and adolescents in Jimma town had attention deficit hyperactivity disorder. Therefore, the prevalence of attention deficit hyperactivity disorder was high. For this reason, there is a need to pay increased attention to control associated factors of attention deficit hyperactivity disorder and reduce its prevalence.

**Supplementary Information:**

The online version contains supplementary material available at 10.1186/s12888-023-04636-9.

## Introduction

Attention-Deficit/Hyperactivity Disorder (ADHD) is a neuropsychiatric disorder affecting pre-school teenagers, children, and adolescents around the world. ADHD is characterized by reduced sustained attention and hyperactivity that interferes with functioning or development [1]. The three basic forms of ADHD described in the Diagnostic and Statistical Manual of mental disorder, Fifth Edition (DSM-5) are: predominantly inattentive (ADHD-I), hyperactive/impulsive (ADHD-H), and combined type (ADHD-C) [2]. Of those three subtypes ADHD-I describes individuals with maladaptive levels of inattention, but not ADHD-H, while ADHD-H type is characterized by changes in levels of hyperactivity-impulsivity, and the ADHD-C type is individuals with symptoms of both inattention and hyperactivity-impulsivity [3]. The onset of ADHD symptoms should be before age 12 and to make a diagnosis six or more symptoms of inattention or hyperactivity impulsivity type that should lead to significant impairment in multiple settings [4]. Although ADHD can be diagnosed among adults, its most often identified when children first start school which allows for early interventions to minimize social and educational disabilities related to this disorder [5].

Globally, ADHD prevalence estimates are 5.9–7.1% in children and adolescents depending on the source of information for the diagnosis [6]. From a national survey among US children aged 4 to 17 years, the prevalence rate of ADHD was 11% and this has increased by 42% from 2003 to 2011 [7]. In a similar manner, in US alone it affects about 2 million children; on average, at least one child in each classroom needs assistance in the event of a disorder [8]. The prevalence of ADHD symptoms in Arab countries varies considerably between 1.3 and 16% [9] and Studies conducted in Africa were limited but certain studies reported prevalence of ADHD which ranges between 5.4% and 8.7% among school children and 1.5% in the general population [10].

Children with ADHD have many challenges that include lack of attention, and poor judgment on the outcome of their actions [11], poor academic record, and difficulty in following instructions [12]. Moreover, children with ADHD are more likely than their peers to experience frequent injury, higher rates of co-occurring psychiatric disorders, and greater healthcare utilization [13]. ADHD can persist into adulthood and have a significant impact across many aspects of a patient’s life including: social, academic, occupational functioning, and quality of life [14]. In addition to the patient’s life, ADHD can have an impact on the lives of parents, caregivers, and other family members by increasing problems at home and straining family connections [15]. But early recognition, assessment, and management of children with ADHD can redirect their educational and psychosocial development problems compared to interventions that can be initiated late during adulthood [16]. The National Institute of Health (NIH) described ADHD as a major health cost and reported that public school expenditures for children diagnosed with ADHD have averaged between 3.5 and 4 billion USD annually in the United State and with an estimated annual societal cost of $34 billion to $52 billion [17]. ADHD is among the 15 leading causes in children aged 5–19 years according to the global burden of diseases (GDB) study of 2010 which was the first to include ADHD for burden quantification indicated that globally, year life disability/disability-adjusted life year (YLDs/DALYs) was 491,500 and accounted for (0.80%) of total global YLDs and 0.25% of total global DALYs [18]. Considering the significant burden of disease, early identification and treatment interventions alone are not enough to reduce the prevalence and economic costs of this disorder. As a result, it would be beneficial to look at contributing factors that may predict ADHD.

The etiology of ADHD is not known, however, it is a result of a complex interaction between genetic, environmental, developmental traits, and genetic factors. About 80% of ADHD are due to genetic factors [19]. A previous study from United State indicated that TV usage, participation in sports, two-parent family structure, and family members’ smoking status were significantly associated with ADHD [20]. Other researchers from Egypt also reported associations between ADHD and factors like a low socioeconomic level, higher birth order, male gender, smoker fathers, consanguineous parents, family history of ADHD, low birth weight, artificially fed children, and pupils living with a single parent [21]. Other factors that have been found to have an association with ADHD include watching TV, cyanosis, and head trauma [22], being male, living with a single parent, childbirth order/rank, and low family socioeconomic status [23]. However, these have not been comprehensively studied in Ethiopia, indicating the importance of conducting further studies to understand these factors and generate evidence that will enable the design of strategies that address ADHD. Additionally, important variables like childhood history of head trauma, maternal complication during pregnancy and child feeding style during first six months were not well addressed in existing literatures indicating the importance of conducting further studies to understand these factors and generate evidence that will enable the design of strategies that address ADHD.

The limited attention given to mental health is reflected by the scarcity of data on ADHD in sub-Saharan Africa, and Ethiopia in particular. Due to increasingly challenging behavioral outcomes, among children with ADHD globally, various studies have been conducted among children and adolescents but there is limited data in Ethiopia. Finally, Although studying ADHD is important to come up with evidence which is important to make an intervention, there was a limited study that investigated this in the study area. Therefore, the findings of this study will help to fill this gap and shows the prevalence and associated factors of ADHD among children aged 6 to 17 years living in Jimma town.

## Methods

### Setting

The study was conducted in Jimma town from located in Western Oromia regional state, Southwest Ethiopia. The town is found 351 km to the Southwest of Addis Ababa, the capital city of Ethiopia and located at 7º 4‟ north latitude and 36 º 5‟ East longitude and the climatic conditions is “Wayne Dega”. Based on figures from the Central Statistical Agency in 2005, this town has an estimated total population of 159 009, of whom 80 897 were males and 78 112 were females. The town has 17 Kebele and the study was conducted in five kebele [24].

### Study design

A cross-sectional study design was conducted from one August 2021 to one September 2021.

### Participants

**Figure 1:**
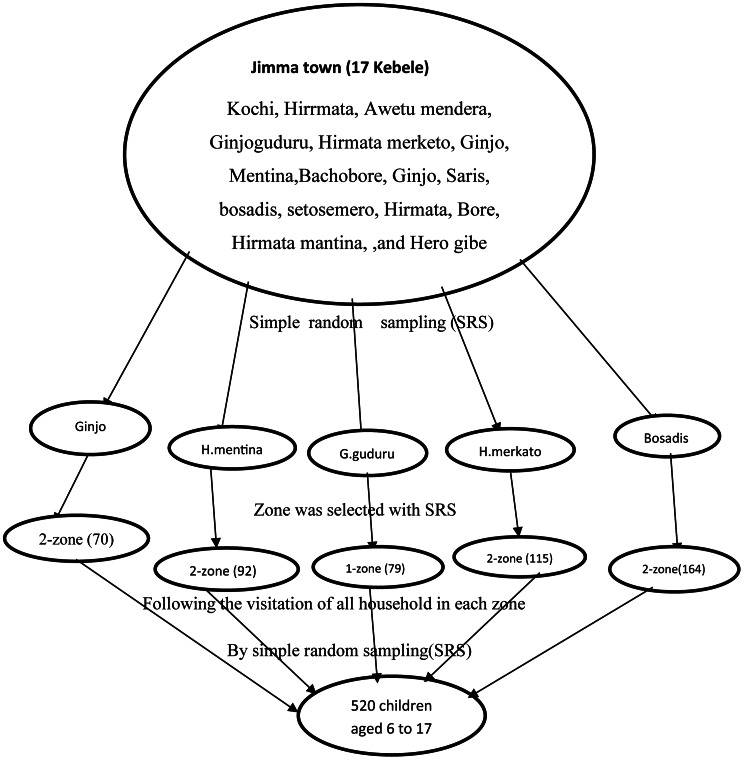
Schematic representation of sampling procedure

The source population of this study was children aged 6 to 17 years who reside in Jimma town, Southwest Ethiopia. Study population was a sample of children between the age of 6 to 17 years from the source population, who fulfils the eligibility criteria. Children aged 6 to 17 years’ old who can able to speak, and had either parents or caregivers All children aged 6 to 17 year who were resident in Jimma town for at least six months. Children and parents who were critically ill during data the collection period and not able communicate. The sample size was determined by using single proportion formula and factors associated with ADHD among children aged 6–17 year from previous research which was conducted in Ethiopia [23]. Calculated sample sizes for risk factors varied between 84 (i.e. single-parent household) to 520 (i.e. being first born) while 358 children would be required to evaluate the prevalence of ADHD among children with 95.0% confidence interval and 10.0% non-response rate from single population propotion formula [23]. The variable child birth order maximizes sample size; therefore, the final sample size needed was 520. A multistage sampling technique was employed. There were seventeen kebele (the smallest administrative unit) in the town. Out of the 17 Kebele five kebele was selected by simple random sampling. Then one to two zones from each selected kebele was selected by simple random sampling and sample was proportionally allocated to each selected zone. After survey was conducted at each selected zone and 2351 fresh list of house number which has at least one child between 6 and 17 years old, in the selected zone was prepared. By using simple random sampling method households was identified in each of the selected zone. If more than one child in a household, one was chosen by the lottery method and parent/caregiver of the the child was interviewed (Fig. 1).

### Study tool and measurements

The presence of ADHD among children aged 6 to 17 years old were assessed by using the Vanderbilt ADHD diagnostic parent rating scale (VADHD) based on the DSM-IV criteria of ADHD on the original scoring. The scale consists of 18 items representing symptoms of ADHD, and the VADHD rating scale is a proxy-administered (parent or teacher) questionnaire that is based on the Diagnostic and Statistical Manual of Mental Disorders, 4th Edition (DSM-IV) diagnostic criteria for ADHD. Each symptom were rated on a 4-point likert type scale indicating the occurrence and the severity of symptoms: 0 (never), or (rarely), 1 (sometimes), 2 ( often) and 3 (very often ). The diagnostic criteria for ADHD were met if the informants marked six items or more for any subtype (Inattentive or Hyperactivity/Impulsivity) as “often” or “very often.” Inattention requires six or more counted behaviors from questions 1 to 9 as“often” or “very often.” an indication of the predominantly inattentive subtype. Hyperactivity-impulsive requires six or more counted behaviors from questions 10 to 18 as“often” or “very often.” an indication of the predominantly hyperactive/impulsive subtype. Combined requires six or more counted behaviors each from both the subtype inattention and sub-type hyperactivity/impulsivity dimensions.Vanderbilt ADHD diagnostic parent rating scale has performance sections as impairment criteria in addition to symptoms criteria. The scale consists of 8 items representing impairments. The Performance scale were rated on five point likert scale indicating the imparment criteria: excellent [1], above-average [2], average [3] somewhat of a problem [4] problematic [5]. Children who had acquired scores of six or more on 18 items and score a [4] or [5] on any of eight items on performance questions was considered as having ADHD [25].

The VADHD rating Scale was pretested for validity in our sample and was found to be easily understood by the participants with internal consistency (Cronbach’s alpha = 0.94). Data was collected from parents using face to face interview technique using the VADHD Rating scale items that was translated to the local language Afaan Oromo and Amharic. A structured questionnaire of possible associated factors related to ADHD were attached to the parent’s rating scale which was filled at home. In addition, potential explanatory variables identified by reviewing literature included the following Sociodemographic factors: information regarding both children and their parents’ age, sex, educational level of father/mother, occupation of father/mother, monthly family income, child feeding style during first six months: “Was this child breastfed or bottle feeding during his/her first six months,” yes/no, head injury with loss of consciousness yes/no, mother’s age at time of the pregnancy, family history of mental illness yes/no, complications during labor/delivery Yes/No If yes what complication?, alcohol exposure during pregnancy yes/no, nicotine exposure during pregnancy yes/no, and information regarding parent marital status: married, divorced/separated/widowed. Family size: based on the Ethiopia Demographic and Health Survey (EDHS), those urban households who have family size of ≥ 4 persons was considered as having above average family size and family monthly income: Using the world bank poverty line cut point those family who have average monthly income of less than 2,565 ETB(1.9$/day) was taken as below poverty line.

A structured questionnaire was used; it was translated to the local language Afaan Oromo and Amharic by experts in three languages and translated back to English by another person to ensure consistency and accuracy; before data collection training was given for data collectors. Five BSc Psychiatry and five clinical nurse were participated in data collection. After ethical review board approval, the investigator visited Jimma town health office and explained the aims and methods of the study to the office then permission to conduct the study was obtained. A latter of invitation to participate in the study was be given to the children’s parents and the latter explain the aims and methods of the study, name and contact information of the chief researcher, and that participation was voluntary. The questionnaire were prepared first in English and translated into Afaan Oromo/Amharic language then back translated to English to check consistency. Training was given for data collectors, and supervisor. Pre-test was conducted (5% of the sample size) at Agaro town to identify potential problems in data collection tools and modification of the questionnaire. Regular supervision and support were given for data collectors by the supervisors and principal investigator. Data were checked for completeness and consistency by supervisors and principal investigator on daily bases during data collection time. The anonymity and confidentiality of the information was kept at all stages of data processing.

### Statistical analysis

Data was entered into Epi Data Version 4.6 [26] and analyzed using SPSS version 26 [27]. Descriptive statistics were used to describe the sample characteristics and to assess the prevalence of ADHD. Multicollinearity and Hosmer-lemeshow goodness model fitness was checked. The association between potential variables and the outcome variable were investigated using bi-variable and multivariable logistic regression. The variables with a p-value less than 0.25 during bivariable analysis were selected for the adjusted model.The level of significance was set at a p-value of < 0.05 to determine the association between outcomes variables and possible explanatory variables.

## Results

### Sociodemographic characteristics of children aged 6 to 17 years

A total 520 children were invited to participante in the study and 504 completed the interview with a response rate of 96%. Out of the total children,(56%,n = 282) were males. About half of children (54.2%, n = 273) were between the ages of 12 and 17 years. About half of the parents, (256, n = 50.8%) were Muslim by religion. Likewise, most of the parents (80.6%, n = 406) were married, while (6.2%, n = 31) were divorced/separated. Out of the total mothers, (55.8%, n = 281) attended school until secondary school or above. Regarding family size, (51.4%, n = 259) of the families had more than four children in the house.Three fourth (70.4%, n = 355) of families had a monthly income above poverty line of greater than 2565ETB. About half of children (54.8%, n = 276) were the first child in birth order attending primary school (49.2%, n = 248). Almost all (96%, n = 484) of participants were living with their family (Table 1).

**Table 1:** Socio-demographic and family related characteristics of children in Jimma town, Southwest Ethiopia, September, 2021

	Variables	Frequency		Percent(%)
Sex	Male	282		56.0
	Female	222		44.0
Age	6–11	231		45.8
	12–17	273		54.2
Level of education	Illiterate	12		2.4
	able to read and write	4		0.8
	primary school	248		49.2
	secondary school and above	240		47.6
Birth order	first child	276		54.8
	second child and above	228		45.2
living circumstance of parent’s	both are alive	422		83.7
	only mother alive	67		13.3
	only father alive	10		2.0
	both are died	5		1.0
With whom the child is living	with family	484		96.0
	Relative	20		4.0
Father’s occupation	government employee	167		33.1
	Merchant	150		29.8
	Farmer	50		9.9
	daily laborer	76		15.1
	Others	61		12.1
mother’s occupation	house wife	243		48.2
	Merchant	79		15.7
	government employee	137		27.2
	daily laborer	45		8.9
mother’s educational status	unable to read and write	81		16.1
	primary school	142		28.2
	secondary school and above	281		55.8
Father’s educational status	unable to read and write	27		5.4
	primary school	156		31.0
	secondary school and above	321		63.7
family size	1–4	245		48.6
	greater than 4	259		51.4
Parent marital status	Married	406		80.6
	divorced/separated	31		6.2
	Widowed	67		13.3
family type	nuclear family	471		93.5
	extended family	33		6.5
Maternal age during pregnancy	17–24	156		31.0
	25 and above	348		69.0
Family monthly income	Below poverty line	149	29.6	
	Above poverty line	355		70.4

### Clinical, obstatric and neonatal factors of children aged 6–17 year

From a total of 504 children participated on the study; (53, 10.5%) had reported maternal complication during pregnancy such as, bleeding (8.3%), hypertension (5.0%) and others (4.2%). whereas 37(7.3%) had family history mental illness, 116(23%) were preterm birth, 54(10.7%) had some kind of complication during delivery, 37(7.3%) had history of head trauma, and 43(8.5%) had bottle feeding during first six month (Table 2).

**Table 2:** Clinical, obstatric and neonatal factors of children aged 6–17 year in Jimma town, Southwest Ethiopia, September, 2021

Variables		Frequency	Percent(%)
Maternal complication during pregnancy	Yes	53	10.5
family history of mental illness	Yes	37	7.3
Mother’s exposure to insecticide or herbicide during pregnancy	Yes	12	2.4
Maternal health status during pregnancy	Sick	56	11.1
duration of pregnancy	Preterm	116	23.0
complication at delivery	Yes	54	10.7
child cried soon after delivery	Yes	300	59.5
Child feeding style during first six month	breast feeding	461	91.5
	bottle feeding	43	8.5
history of head trauma for child	Yes	37	7.3
Child’s chronic physical illness	Yes	28	5.6

Notes: Other complication during pregnancy, hyperemesis

### Substance related factors

**Fig. 2:**
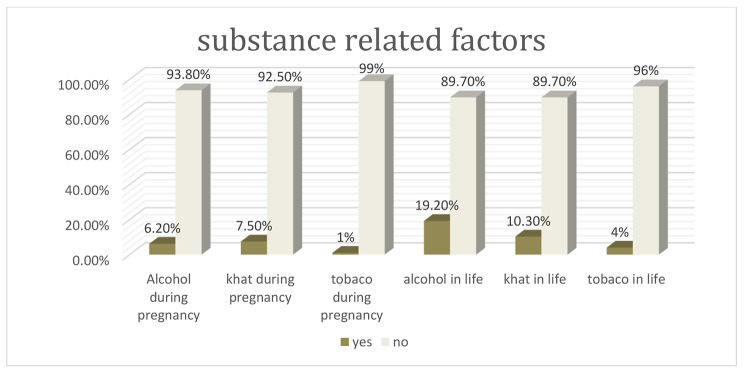
Distribution of different types of substances used among mother’s of study participants in Jimma town, Southwest Ethiopia, 2021

Out of the total mothers, (14.7%, n = 74 ) used substance during pregnancy. Likewise, (7.5%, n = 38) mothers reported they have used khat, while (1.0%, n = 5) and (6.20%, n = 31) reported cigarrete and alcohol use respectively. Regarding mother’s ever use of substance (4.0%, n = 21) mothers reported they have used tobacco, (10.3%, n = 52) used khat and (19.2%, n = 97) used alcohol (Fig. 2).

### Prevalence of attention deficit hyperactivity disorder

The prevalence of ADHD among children aged 6–17 year was (9.9%, n = 50) 50(9.9%) with 95% CI (7.3, 12.54).

### Factors associated with attention deficit hyperactivity disorder

In the bivariable analysis male gender, age 6–11 years, low mother educational status, father’s educational status, child birth order, family monthly income below poverty line, mother’s occupational status, father’s occupational status, young maternal age during pregnancy, family size, child-health status before six years, maternal health status during pregnancy, parental substance use, duration of pregnancy, complication at delivery, history of head trauma, child feeding style during first six month, complication during pregnancy and time spent in watching television were found to be associated with ADHD and entered to multivariate analysis (**Table S 1**). After controlling confounders using multivariable binary logistic regression, young age 6–11 years, maternal complication during pregnancy, history of head trauma, child feeding style during first six month, alcohol use during pregnancy, and mother’s educational status were associated with attention deficit hyperactivity disorder. Younger age 6–11 year was about four times more likely to have ADHD than age 12–17 year (AOR = 3.86, 95% CI=(1.77–8.43)). Additionally, the odds of having ADHD was 3.6 fold higher (AOR = 3.56, 95% CI = 1.44–8.79) among children and adolescent who had maternal complication during pregnancy as compared with those who have no maternal complication during pregnancy.This study has revealed that children aged 6–17 year who have head trauma had more than three times more likely to have ADHD (AOR = 3.27, 95% CI = 1.294, 8.262) than those who have no history of head trauma. Additionally, children and adolescent who have maternal alcohol use during pregnancy were three times more likely to have ADHD than those who have no maternal alcohol use during pregnancy (AOR = 3.30, 95% CI = 1.12, 9.69). Likewise, bottle feeding during first six months increases the likelihood of having ADHD by about three times as compared to children and adolescents who were fed breast, (AOR = 2.94, 95% 1.23, 7.03). Children who have low maternal education(unable to read and write) and was about more than three times more likely to have ADHD than secondary school and above (AOR = 3.10, 95% CI = 1.24–7.79). Similarly, children and adolescents whose mother have primary education were about 3-times more likely to have ADHD than secondary school and above (AOR = 2.97, 95% CI = 1.32–6.73) (Table 3).

**Table 3:** multivariable logistic regression of factors associated with ADHD of children aged 6–17 year in Jimma town, Southwest Ethiopia, 2021

Variables	ADHD		AOR & 95% CI	P Value
	Yes N %	No N %		
Child’s age 6–11 12–17	39(16.9) 11(4.0)	192(83.1) 262(96.0)	3.86(1.77–8.43) 1	0.001* 1
mother’s educational status unable to read and write primaryschool secondary school	19(23.5) 19(13.4) 12(4.3)	62(76.5) 123(86.6) 269(95.7)	3.10(1.24–7.79) 2.97(1.32–6.73) 1	0.016* 0.009* 1
complication during pregnancy No Yes	418(92.7) 36(67.9)	33(7.3) 17(32.1)	1 3.56(1.44–8.79)	1 0.006*
Head trauma for children No Yes	434(92.9) 20(54.1)	33(7.1) 17(45.9)	1 4.2(1.68–10.37)	1 0.002*
Child feeding style 1st 6 month Breast feeding Bottle feeding	421(93.1) 33(63.5)	31(6.9) 19(36.5)	1 3.56(1.53–8.29)	1 0.003*
Alcohol use during pregnancy No Yes	432(91.3) 22(71.0)	41(8.7) 9(29.0)	1 3.54(1.26-10)	1 0.017*

Notes: * Factors that have association at p-value < 0.05 1 = reference category AOR = Adjusted odds ratio

## Discussion

This study assessed the prevalence and associated factors of ADHD among children and adolescents in Jimma town Oromia regional state, Southwest Ethiopia. Overall, 9.92% of children and adolescents were found to have ADHD reflecting an increase in prevalence, is an issue of public health concern. This findings also indicate that child’s age 6–11 year, maternal complication during pregnancy, history of head trauma, bottle feeding during first six months, alcohol use during pregnancy, and low maternal education were found to have strong association with ADHD.

The prevalence of ADHD in this study was similar to previous Ethiopian study that was conducted in Guji zone of Girja District (7.3%) [23] and findings from other countries that were conducted in Nigeria (8.7%) [28], Egypt (9.6%) [21], Jeddah city of Saudi Arabia (12.3%) [29], India (11.3%) [30], Venezuela (10%) [31], and United state (10%) [20]. However, it was higher than the studies conducted in Kenya (6.3%) [32], Nigeria (4.7%) [33], Thailand (2.2%) [34], Brazil (5.8%) [35], and Spain (6.8%) [36], but lower than another previous studies from Ethiopia (13.7) [37], and Egypt (21.8%) and (16.2%) according to teacher and parent rating scale respectively [38].

The higher prevalence of ADHD in the current study compared to the previous study from Kenya might be due to the difference in the study setting, difference in age range of study participants and sample size. As study from Kenya was conducted at health institution that was a tertiary care hospital accident and emergency unit and included 240 children aged 6 to 12, whereas the present study is community-based which includes 520 children aged 6 to 17 years. Furthermore, differences between the Nigerian study and our study might be explained by differences in data collection tools employed, as they used DBDRS, just symptoms criterion, and their age ranged from 5 to 12 years. Additionally, the difference between Thailand’s study and ours is that the former was performed in a school setting, and they employed SNAP-IV to collect data and the diagnosis was validated by a pediatric neuropsychiatrist using DSM-5 criteria, however, we used the Vanderbilt ADHD rating scale for this community based study.

Furthermore, the lower prevalence of ADHD in the current study compared to study conducted in rural district of Ethiopia might be attributed to the difference in study setting and Data collection tools as they did in a rural districts, and they used DBDRS, just symptoms criterion, to collect data however, our study was conducted in urban population, we used Vanderbilt ADHD rating scale with the requirement of impairment criteria for the diagnosis. Additionally, the difference between Egypt study and ours is that the former was performed in both a school setting and home setting, and they used DSM-5 symptom criteria to collect data. However, we used the Vanderbilt ADHD rating scale with the inclusion of impairment Criterion which has been found to be significantly alter the prevalence of ADHD (all types) and at home setting only. As previous evidence has indicated [39], diagnostic criteria, impairment criterion and source of information associated with differences in prevalence estimate of ADHD which might lead to the lower prevalence of ADHD in the current study.

The current study indicated that children who had head trauma had higher odds to have ADHD compared to those who were not with head trauma, that was consistent with previous study [22]. Furthermore, a previous evidence indicated that the proportion of head injury such as “fracture of skull, neck and trunk; intracranial injury was three times more common in children with ADHD [3]. One of the possible explanation for this association is the occurrence of minor damage in the central nervous system, which can lead to the appearance of ADHD in those children [38].

Bottle feeding also had increased odds of experiencing ADHD, the association between bottle feeding and ADHD was supported by previous studies conducted in Egypt and Saudi arabia [22, 40] where bottle-feeding was significantly higher among cases than controls.This might be linked to a lack of warmth and interaction with the mother when breast-feeding in addition to the neurodevelopmental benefits of breast feeding.

Low level of the moternal education also had higher odds to have ADHD in children and adolescents. This was inline with the previous study conducted in Uganda that a child whose primary caretaker had either no education or had primary education were more likely to have ADHD compared highest level of education [41].This was also consistent with studies conducted in Dammam Saudi Arabia, and Egypt [42, 43]. This might be also explained by the possibility that the children’s caregiver had undiagnosed ADHD in childhood which had a detrimental influence on their educational success. In a review of ADHD, Biederman et al. reported that 5–66% of children with ADHD continue to have the disease into adulthood and that parents of children with ADHD were more likely to have ADHD [44].

This study also observed that age less than 11 years had higher odds to have ADHD. This was similar to the study conducted among Ugandan children; in which Children less than 10 years were more likely to have ADHD [41].This finding also agrees with Biederman et al. who observed a decline in ADHD symptoms with increasing age among different age groups of children with ADHD for 4 years [45]. The possible explanation tor this association is as age increase the brain maturity also increase and children started to regulate themselves. Additionally, ADHD symptom is noticeable at a young age and may become more noticeable when the child start school. Similarly, the majority of symptoms are identified between the ages of 6 and 12 years old, and the symptoms of ADHD usually improves with age [3].

These results indicate that, maternal complication during pregnancy and alcohol use during pregnancy had higher odds to have ADHD, which was consistent with a study conducted in Uganda, which found that a maternal complication during pregnancy was significantly associated with ADHD [41]. Another study that supports this finding was a case-control study conducted in Brazil, which revealed that maternal complication during pregnancy was significantly associated with ADHD [46]. A possible explanation for the link between maternal complications during pregnancy and ADHD is that maternal complications might cause maternal stress, which can affect fetal neurodevelopment and predispose to ADHD [46, 47]. This might be also explained by the role of genetic factors, because of about 80% of ADHD are due to genetic factors [19]. The association between ADHD and alcohol use during pregnancy was inline with study done by the Norwegian Institute of Public Health, US and Beirut [48–50]. This could be explained by the possibility that Alcohol is widely recognized as a teratogenic agent causing CNS dysfunction and impaired mental functioning, including fetal alcohol effect and fetal alcohol syndrome, which incorporates the core symptoms of ADHD [51].

### Strength and Limitations

The study was community-based and included children and adolescents of various ages, increasing its repressentiveness for all children and adolescents in the study context. The most important variables, such as alcohol consumption throughout pregnancy, pregnancy complications, and childhood health condition, were addressed. Standardized instruments were used, and in addition to the symptoms criteria, the impairment criteria were included, which had not been included in the previous studies. Recall bias may exist for child health status before to the age of six, child feeding style in the first six months of life, mother health status during pregnancy, and birth complications.We tried to mitigate this by training interviewers to explain participants the aim of the study, interviewing them in isolated area to maintain their privacy and informing them as their response was anonymous.

## Conclusion

The prevalence of ADHD among children and adolescent is high; one in ten children and adolescent have ADHD in Jimma town. It underlines the importance of greater epidemiological research in this area for treatment and policy implications. A number of factors were significantly associated with ADHD. For this reason, there is a need to pay increased attention to control associated factors of ADHD and reduce its prevalence. Further, there is a need to conduct longitudinal study to investigate the cause effect relationship of risk factors of ADHD. Thus, it was also recommended for future researches to assess ADHD including teacher rating scale, academic performance of school age children and burden of ADHD on academic performance.

## Electronic supplementary material

Below is the link to the electronic supplementary material.

Supplementary Material 1: A bivariable binary logistic regression model of factors associated with ADHD among children aged 6–17 year in Jimma town, Southwest Ethiopia, 2021.

## Data Availability

To protect the anonymity of the participants, the data generated and analyzed during the current study are not publicly available. Upon reasonable request, materials may be obtained from the corresponding author.

## Abbreviations

ADHD: Attention deficit hyperactivity disorder
ADHD-C: Attention deficit hyperactivity disorder combined type
ADHD-H: Attention deficit hyperactivity disorder hyperactive type
ADHD-I: Attention deficit hyperactivity disorder impulsive type
VADHDRS: Vanderbilt Attention deficit hyperactivity disorder rating scale
CDC: communicable diseases control
DSM: Diagnostic statistical manual of mental disorder
ETB: Ethiopian birr
GDB: global burden of diseases
ICD-10: international classification of diseases
NIH: national institute of health
US: United States USD:United State of American dollars
USD: United State of American dollars

## References

[1] Benjamin J, Sadock et al. S. Kaplan & Sadok’s comprehensive Text book of Psychiatry. 2017. 9141–9155 p.

[2] American Psychiatric Association, Diagnostic and Statistical Manual of Mental Disorders: DSM-5, American Psychiatric Association, Washington, DC., 2013. 2013. 2454 p.

[3] Moriyama TS, Cho M, Verin AJ, Fuentes RE, Polanczyk J, Verin GV. MIPH RE. IACAPAP Textbook of Child and Adolescent Mental Health SECTION D EXTERNALIZING DISORDERS. 2009. 6–7 p.

[4] Chiche S. DSM-5. In: Troubles mentaux et psychothérapies. 2016. p. 38.

[5] Willcutt EG (2012). The prevalence of DSM-IV Attention-Deficit/Hyperactivity disorder: a Meta-Analytic Review. Neurotherapeutics.

[6] Rocco I, Corso B, Bonati M, Minicuci N (2021). Time of onset and/or diagnosis of ADHD in european children: a systematic review. BMC Psychiatry.

[7] Visser SN, Danielson ML, Bitsko RH, Holbrook JR, Kogan MD, Ghandour RM et al. Trends in the parent-report of health care provider-diagnosed and medicated attention-deficit/hyperactivity disorder: United States, 2003–2011. J Am Acad Child Adolesc Psychiatry [Internet]. 2014;53(1):34–46.e2. Available from: 10.1016/j.jaac.2013.09.001 10.1016/j.jaac.2013.09.001 PMC4473855 24342384

[8] Klassen AF, Miller A, Fine S, Klassen AF, Miller A, Fine S (2004). Health-Related Quality of Life in Children and Adolescents who have a diagnosis of attention-deficit / hyperactivity disorder the online version of this article, along with updated information and services, is located on the world wide web at : Health-R. Am Acad Pediatr.

[9] Alhraiwil NJ, Ali A, Househ MS, Al-Shehri AM, El-Metwally AA (2015). Systematic review of the epidemiology of attention deficit hyperactivity disorder in arab countries. Neurosciences.

[10] Bakare MO (2012). Attention deficit hyperactivity symptoms and disorder (ADHD) among african children: a review of epidemiology and co-morbidities. Afr J Psychiatry.

[11] Centers for Disease Control and Prevention (CDC). Increasing prevalence of parent-reported attention-deficit/hyperactivity disorder among children United States, 2003 and 2007. MMWR Morb Mortal Wkly Rep [Internet]. 2010;59(44):1439–43. Available from: http://www.ncbi.nlm.nih.gov/pubmed/21063274 21063274

[12] Barkley RA. Attention-Deficit Hyperactivity Disorder. Semin Pediatr Neurol [Internet]. 2006;13(4):279–85. Available from: https://www.jstor.org/stable/10.2307/26057943%0AJSTOR 10.1016/j.spen.2006.09.008 17178358

[13] Danielson ML, Visser SN, Chronis-Tuscano A, DuPaul GJ. A National Description of Treatment among United States Children and Adolescents with Attention-Deficit/Hyperactivity Disorder. J Pediatr [Internet]. 2018;192:240–246.e1. Available from: 10.1016/j.jpeds.2017.08.040 10.1016/j.jpeds.2017.08.040 PMC5732840 29132817

[14] Harpin VA (2005). The effect of ADHD on the life of an individual, their family, and community from preschool to adult life. Arch Dis Child.

[15] Brod M, Pohlman B, Lasser R, Hodgkins P (2012). Comparison of the burden of illness for adults with ADHD across seven countries: a qualitative study. Health Qual Life Outcomes.

[16] Comittee on Quality Improvement, Subcomittee on ADHD (2000). Clinical practice Guideline: diagnosis and evaluation of the child with. Pediatrics.

[17] Larson K, Russ SA, Kahn RS, Halfon N (2011). Patterns of comorbidity, functioning, and service use for US children with ADHD, 2007. Pediatrics.

[18] Erskine HE, Ferrari AJ, Polanczyk GV, Moffitt TE, Murray CJL, Vos T (2014). The global burden of conduct disorder and attention-deficit / hyperactivity disorder in 2010. J ofChild Psychol Psychiatry.

[19] Mick E, Faraone SV, Genetics of Attention Deficit Hyperactivity Disorder. Child Adolesc Psychiatr Clin N Am [Internet]. 2008;17:261–84. Available from: 10.1016/j.chc.2007.11.011%0Achildpsych.theclinics.com 10.1016/j.chc.2007.11.011 18295146

[20] Lingineni RK, Biswas S, Ahmad N, Jackson BE, Bae S, Singh KP (2012). Factors associated with attention deficit / hyperactivity disorder among US children: results from a national survey. Lingineni al BMC Pediatr.

[21] Ahmed SM (2018). Attention deficit hyperactivity disorder in a rural area of Sohag Governorate. Egypt J Community Med.

[22] El-gendy SD, El-bitar EA, El-awady MA, Hanaa E (2017). Attention-Deficit/Hyperactivity disorder: prevalence and risk factors in egyptian primary School Children. Egypt J Community Med.

[23] Lola HM, Belete H, Gebeyehu A, Zerihun A, Yimer S, Leta K (2019). Attention deficit hyperactivity disorder (ADHD) among children aged 6 to 17 Years Old living in Girja District, Rural Ethiopia. Behav Neurol.

[24] Agency CS, Ababa A. Ethiopia Demographic and Health Survey. 2005.

[25] Wolraich ML, Lambert W, Doffing MA, Bickman L, Simmons T, Worley K (2003). Psychometric Properties of the Vanderbilt ADHD diagnostic parent rating scale in a Referred Population. J Pediatr Psychol.

[26] Lauritsen JM, Bruus M. EpiData Entry version 4.6. A comprehensive tool for validated entry and documentation of data.EpiData Assoc Odense Denmark, Version. 2018;4(1.0).

[27] Statistics IS, IBM, Corp. Released 2013. IBM SPSS Statistics for Windows, Version 26.0. Armonk, NY: IBM Corp. Google Search. 2013

[28] Famuyiwa AO. O. AO. Attention deficit hyperactivity disorder among Nigerian primary school children Prevalence and co-morbid conditions.Eur Child Adoles cpsychiatry. 2007;(16):10–5. 10.1007/s00787-006-0569-9 17136303

[29] Homid M, Obaidat Y, Hamaidi D (2013). Prevalence of attention deficit and hyperactivity disorder among primary School students in Jeddah city. Life Sci J.

[30] Ajinkya S, Kaur D, Gursale A, Jadhav P (2012). Prevalence of parent-rated attention deficit hyperactivity disorder and associated parent-related factors in primary school children of Navi Mumbai - A school based study. Indian J Pediatr.

[31] Montiel C, Peña JA, Montiel-Barbero I, Polanczyk G (2008). Prevalence rates of attention deficit/hyperactivity disorder in a school sample of venezuelan children. Child Psychiatry Hum Dev.

[32] Wamithi S, Ochieng R, Njenga F, Akech S, Macharia WM (2015). Cross-sectional survey on prevalence of attention deficit hyperactivity disorder symptoms at a tertiary care health facility in Nairobi. Wamithi al Child Adolesc Psychiatry Ment Heal 91.

[33] Oke O, Oseni S, Adejuyigbe E, Mosaku S (2019). Pattern of attention deficit hyperactivity disorder among primary School children in Ile–Ife, South–West, Nigeria. Niger J Clin Pract.

[34] Sakboonyarat B, Chokcharoensap K, Sathuthum N, Chutchawalanon S (2018). Prevalence and Associated factors of attention deficit hyperactivity disorder (ADHD) in a Rural Community, Central Thailand : a mixed methods study. Glob J Health Sci.

[35] Kieling C, Baker-henningham H, Belfer M, Conti G, Ertem I, Omigbodun O (2011). Global Mental Health 2 child and adolescent mental health worldwide: evidence. Lancet.

[36] Catalá-López F, Peiró S, Ridao M, Sanfélix-Gimeno G, Gènova-Maleras R, Catalá MA. Prevalence of attention deficit hyperactivity disorder among children and adolescents in Spain: A systematic review and meta-analysis of epidemiological studies.BMC Psychiatry. 2012;12. 10.1186/1471-244X-12-168 PMC3534011 23057832

[37] Murugan R, Tiruneh F, Therese Maria, PREVALENCE OF ATTENTION DEFICIT HYPERACTIVITY DEVELOPMENTAL DISORDER AMONG CHILDREN IN JIMMA ZONE (2016). OROMIA REGION, SOUTH WEST. Int J Curr Res.

[38] Bishry Z, Ramy HA, El-Sheikh MM, El-Missiry AA, El-Missiry MA (2013). Risk factors for attention deficit hyperactivity disorder in a sample of egyptian adolescents: a case-control study. Middle East Curr Psychiatry.

[39] Polanczyk GV, Willcutt EG, Salum GA, Kieling C, Rohde LA. Original article ADHD prevalence estimates across three decades: an updated systematic review and meta-regression analysis.Int J ofEpidemiology. 2014;(January):434–42. 10.1093/ije/dyt261 PMC4817588 24464188

[40] Al Hamed JH, Taha AZ, Sabra AA, Bella H. Attention Deficit Hyperactivity Disorder (ADHD) among Male Primary School Children in Dammam, Saudi Arabia: Prevalence and Associated Factors. J Egypt Public Health Assoc [Internet]. 2008;83(3–4):165–82. Available from: http://www.ncbi.nlm.nih.gov/pubmed/19302773 19302773

[41] Wamulugwa J, Kakooza A, Kitaka SB, Nalugya J, Kaddumukasa M, Moore S (2017). Prevalence and associated factors of attention deficit hyperactivity disorder (ADHD) among ugandan children; a cross – sectional study. Child Adolesc Psychiatry Ment Health.

[42] Alizadeh H, Applequist KF, Coolidge FL (2007). Parental self-confidence, parenting styles, and corporal punishment in families of ADHD children in Iran. Child Abus Negl.

[43] Ouyang L, Fang X, Mercy J, Perou R, Grosse SD (2008). Attention-Deficit/Hyperactivity disorder symptoms and child maltreatment: a Population-Based study. J Pediatr.

[44] Biederman J (2005). Attention-deficit/hyperactivity disorder: a selective overview. Biol Psychiatry.

[45] Biederman J, Mick E, Faraone SV (2000). Age-dependent decline of symptoms of attention deficit hyperactivity disorder: impact of remission definition and symptom type. Am J Psychiatry.

[46] Ketzer CR, Gallois C, Martinez AL, Rohde LA, Schmitz M (2012). Is there an association between perinatal complications and attention-deficit/hyperactivity disorder-inattentive type in children and adolescents?. Rev Bras Psiquiatr.

[47] Kessler RC, Ph D, Adler L, Barkley R, Ph D, Biederman J (2006). The prevalence and correlates of adult ADHD in the United States: results from the National Comorbidity Survey Replication. (Am J Psychiatry.

[48] Ghossoub E, Ghandour LA, Halabi F, Zeinoun P, Shehab AAS, Maalouf FT (2017). Prevalence and correlates of ADHD among adolescents in a Beirut community sample: results from the BEI-PSY study. Child Adolesc Psychiatry Ment Health.

[49] Eilertsen EM, Gjerde LC, Reichborn-Kjennerud T, Ørstavik RE, Knudsen GP, Stoltenberg C (2017). Maternal alcohol use during pregnancy and offspring attention-deficit hyperactivity disorder (ADHD): a prospective sibling control study. Int J Epidemiol.

[50] Sandler AD (2002). Case-control study of attention-deficit hyperactivity disorder and maternal smoking, alcohol use, and drug use during pregnancy. J Dev Behav Pediatr.

[51] Burger PH, Goecke TW, Fasching PA, Moll G, Heinrich H, Beckmann MW (2011). How does maternal alcohol consumption during pregnancy affect the development of attention deficit/hyperactivity syndrome in the child. Fortschr der Neurol - Psychiatr.

