# Supplementary material for: High rate of attention deficit hyperactivity disorder among children 6 to 17 years old in Southwest Ethiopia findings from a community-based study
Source: BMC Psychiatry. 2023 Mar 8;23:144. doi: 10.1186/s12888-023-04636-9 (PMC9993367; doi:10.1186/s12888-023-04636-9)
Supplement: Supplementary Material 1: — A bivariable binary logistic regression model of factors associated with ADHD among children aged 6–17 year in Jimma town, Southwest Ethiopia, 2021. [file 12888_2023_4636_MOESM1_ESM.pdf]

Table S1: A bivariable binary logistic regression model of factors associated with ADHD among children aged 6-17 year in Jimma town, southwest Ethiopia, 2021

Variable	Categories	ADHD		COR & 95% CI	P Value
		Yes	No		
**child's sex**	Male	33(11.7%)	249(88.3%)	1.60(0.86-2.95)	0.13*
	Female	17(7.7%)	205(92.3%)	1	1
**Child's age**	6-11	39(16.9%)	192(83.1%)	4.83(2.42-9.70)	<0.001*
	12-17	11(4.0%)	262(96.0%)	1	1
**Child's level of education**	unable to read and write	1(7.1%)	13(92.9%)	1	1
	Primary school and above	49(10%)	441(90.0%)	1.44(0.18-11.28)	0.73
**With whom the child is living**	with family	47(9.7%)	437(90.3%)	1	1
	Others	3(15.0%)	17(85.0%)	1.64(0.46-5.80)	0.44
**current living circumstance of parent's**	both are alive	41(10.3%)	359(89.8%)	1	1
	only mother alive	5(6.2%)	76(93.8%)	0.58(0.22-1.51)	0.26
	only father alive	2(20.0%)	8(80.0%)	2.20(0.45-10.66)	0.33
	both are died	2(15.4%)	11(84.6%)	1.59(0.34-7.43)	0.55
**Family size**	1-4	17(6.9%)	228(93.1%)	1	1
	4 and above	33(12.7%)	226(87.3%)	1.958(1.06-3.61)	0.032*
**father's occupation**	government employee	12(7.0%)	160(93.0%)	1	1
	Merchant	16(11.1%)	128(88.9%)	1.667(0.76-3.65)	0.20*
	Farmer	8(16.0%)	42(84.0%)	2.540(0.97-6.61)	0.06*
	daily laborer	11(14.1%)	62(85.9%)	2.19(0.92-5.20)	0.08*

	Others	3(5.0%)	57(95.0%)	0.70(0.19-2.57)	0.59
**Mother's occupation**	house wife	25(10.3%)	218(89.7%)	1	1
	Merchant	5(6.3%)	74(93.7%)	0.59(0.22-1.59)	0.30
	government employee	8(5.8%)	129(94.2%)	0.54(0.24-1.23)	0.144*
	daily laborer	12(26.7%)	33(73.3%)	3.17(1.45-6.91)	.004*
**Father's educational status**	unable to read and write	7(18.9%)	30(81.1%)	3.66(1.92-6.98)	<0.001*
	primary school	26(17.2%)	125(82.8%)	4.10(1.58-10.68)	.004*
	secondary school and above	17(3.37%)	299(59.3%)	1	1
**mother's educational status**	unable to read and write	19(23.5%)	62(76.5%)	6.87 (3.17-14.89)	<0.001*
	primary school	19(13.4%)	123(86.6%)	3.46(1.63-7.36)	.001*
	secondary school and above	12(4.3%)	269(95.7%)	1	1
**Maternal age during pregnancy**	17-24	10(6.4%)	146(93.6%)	0.53(0.26-1.08)	0.08*
	25 and above	40(11.5%)	308(88.5%)	1	1
**child's birth order**	first child	18(6.5%)	258(93.5%)	1	1
	second child and above	32(14.0%)	196(86.0%)	2.34 (1.28-4.29)	0.006*
**Family income**	Below poverty line	21(14.1%)	128(85.9%)	1.84(1.02-3.35)	0.045*
	Above poverty line	29(8.2%)	326(91.8%)	1	1
**parent marital**	Married	40(10.2%)	352(89.8%)	1	1

status	divorced/separated	5(12.5%)	35(87.5%)	1.26(0.47-3.39)	0.65
	Widowed	5(6.9%)	67(93.1%)	0.66(0.25-1.73)	0.39
**family type**	nuclear family	48(10.2%)	423(89.8%)	1	1
	extended family	2(6.1%	31(93.9%)	0.57(0.13-2.45)	0.45
**family history of mental illness**	No	421(90.1%)	4(10.8%)	1	1
	Yes	33(89.2%)	46(9.9%)	1.11 (0.38-3.27)	0.851
**Child-health status before 6 years**	Healthy	393(93.6%)	27(6.4%)	1	1
	Sick	61(72.6%)	23(27.4%)	5.49 (2.96-10.18)	<0.001*
**Maternal complication during pregnancy**	No	418(92.7%)	33(7.3%)	1	1
	Yes	36(67.9%)	17(32.1%)	5.98(3.04-11.77)	<0.001*
**Duration of pregnancy**	Preterm	96(82.8%)	20(17.2%)	2.49(1.35-4.57)	0.003*
	Full term	358(92.3%)	30(7.7%)	1	1
**complication at delivery**	No	417(92.7%)	33(7.3%)	1	1
	Yes	37(68.5%)	17(31.5%)	5.81(2.96-11.40)	<0.001*
**History of head trauma for children and adolescent**	No	434(92.9%)	33(7.1%)	1	1
	Yes	20(54.1%)	17(45.9%)	11.18(5.35-23.36)	<0.001*
**Child feeding style during first six month**	breast feeding	421(93.1%)	31(6.9%)	1	1
	Bottle feeding	33(63.5%)	19(36.5%)	7.82(3.99-15.31)	<0.001*
**Child's chronic physical illness**	No	431(90.5%)	45(9.5%)	1	1
	Yes	23(82.1%)	5(17.9%)	2.08(0.75-5.74)	0.157*

**Maternal health status during pregnancy**	Healthy	402(92.4%)	33(7.6%)	1	1
	Sick	52(75.4%)	17(24.6%)	3.98(2.07-7.65)	<0.001*
**Bleeding**	No	427(92.4%)	35(7.6%)	1	1
	Yes	27(64.3%)	15(35.7%)	6.78(3.30-13.91)	<0.001*
**Pre-eclampsia**	No	433(90.4%)	46(9.6%)	1	1
	Yes	21(84.0%)	4(16.0%)	1.79(0.59-5.45)	0.303
**Diabetes mellitus**	No	447(90.1%)	49(9.9%)	1	1
	Yes	7(87.5%)	1(12.5%)	1.30(0.16-10.81)	0.806
**Sexual transmitted infection**	No	447(90.3%)	48(9.7%)	1	1
	Yes	7(77.8%)	2(22.2%)	2.66(0.54-13.17)	.230*
**Mode of delivery**	spontaneous vaginal delivery	305(93.6%)	21(6.4%)	1	1
	vaginal birth after oxytocin	67(83.8%)	13(16.3%)	2.82(1.34-5.91)	.006*
	Episiotomy	13(72.2%)	5(27.8%)	5.59(1.82-17.16)	.003*
	Instrumental delivery	24(85.7%)	4(14.3%)	2.42(0.77-7.62)	0.131*
	Cesarean section	45(86.5%)	7(13.5%)	2.26(0.91-5.62)	0.08*
**child cried soon after delivery**	No	187(91.7%)	17(8.3%)	0.74(0.40-1.36)	0.33
	Yes	267(89.0%)	33(11.0%)	1	1
**Mother's exposure to insecticide**	No	444(90.2%)	48(9.8%)	1	1
	Yes	10(83.3%)	2(16.7%)	1.85 (0.39-8.69)	0.44

**Ever use of Alcohol**	No	367(90.2%)	40(9.8%)	1	1
	Yes	87(89.7%)	10(10.3%)	1.06(0.51-2.19)	0.89
**Ever use of Khat**	No	417(92.3%)	35(7.7%)	1	1
	Yes	37(71.2%)	15(28.8%)	4.83(2.42-9.65)	<0.001*
**Ever use of Tobacco**	No	440(90.9%)	44(9.1%)	1	1
	Yes	14(70.0%)	6(30.0%)	4.29(1.57-11.71)	0.005*
**Tobacco uses during pregnancy**	No	450(90.2%)	49(9.8%)	1	1
	Yes	4(80.0%)	1(20.0%)	2.30(0.25-20.95)	0.46
**Alcohol uses during pregnancy**	No	432(91.3%)	41(8.7%)	1	1
	Yes	22(71.0%)	9(29.0%)	4.31(1.86-9.97)	0.001*
**khat use during pregnancy**	No	420(91.5%)	39(8.5%)	1	1
	Yes	34(75.6%)	11(24.4%)	3.48(1.64-7.41)	0.001*
**Time spent in TV watching**	Less than 2hrs	181(87.4%)	26(12.6%)	1	1
	2hrs & above	273(91.9%)	24(8.1%)	0.61(0.34-1.09)	0.100*

Notes: Other with whom living now: = relatives, other occupations of father: = private employee, NGO sector employee, and pastor. * Factors that have association at p-value <0.25 1= reference category, COR = Crude odds ratio
